# Pandemic‐resilient target setting in colorectal cancer screening for vulnerable older population

**DOI:** 10.1002/cam4.4907

**Published:** 2022-06-05

**Authors:** Toshiaki Shibata, Daisuke Shinjo, Junichi Takahashi, Kiyohide Fushimi

**Affiliations:** ^1^ Department of Health Policy and Informatics Tokyo Medical and Dental University Graduate School Tokyo Japan; ^2^ Department of Gastroenterology and Hepatology, Medical Hospital Tokyo Medical and Dental University Tokyo Japan

**Keywords:** cancer screening, colorectal cancer, COVID‐19, early detection, pandemic, participation rate

## Abstract

**Background:**

Colorectal cancer screening (CRCS) needs to be pandemic‐resilient to avoid long‐lasting shutdowns; however, realistic participation target remains unelucidated. This study aimed to identify the lowest acceptable participation rate in CRCS during a pandemic, focusing on vulnerable older populations who require urgent intervention.

**Methods:**

This nationwide cross‐sectional study included 80,946 inpatients aged 70–85 years who were first diagnosed with colorectal cancer (CRC) after 70 years of age, between April 1, 2014 and March 31, 2019, in Japan. To evaluate the association between area‐level CRCS participation rate and individual early CRC detection, a multilevel logistic regression model was constructed. The mandatorily implemented screening rates were converted to the total screening rate equivalents (TSREs), which reflect the remaining contributions of voluntarily provided screenings.

**Results:**

Early detections during stages 0–I were significantly observed when primary screening rate was ≥38% (TSRE) and combined follow‐up rate was ≥85%. For early detection during Tis–T1, primary screening rate ≥ 38% (TSRE) and combined follow‐up rate ≥ 90% were necessary. For follow‐up rates ≥70% or ≥75%, there were cases where missed detection of Tis–T1 were observed.

**Conclusion:**

The results indicate that, even during pandemic, CRCS should achieve a primary screening rate of 38% and follow‐up rate of 85% for vulnerable older populations. These values, lower than the current desirable rates, suggest the maximum possible compromise in balancing the resources between cancer screening and pandemic measures. Moreover, they also indicate the minimum target for shifting to fecal immunochemical test‐focused program. Further explorations with varied CRCS settings are necessary for verification.

## Graphical abstract

Colorectal cancer screening requires pandemic‐resilience to avoid the long‐lasting shutdown. This nationwide study, focusing on pandemic‐vulnerable older population, indicates that colorectal cancer screening should achieve a primary screening rate ≥38% and follow‐up rate ≥85%, which serves as a foundation for balancing the resources between cancer screening and pandemic measures or as the minimum target for shifting to fecal immunochemical test focused program.

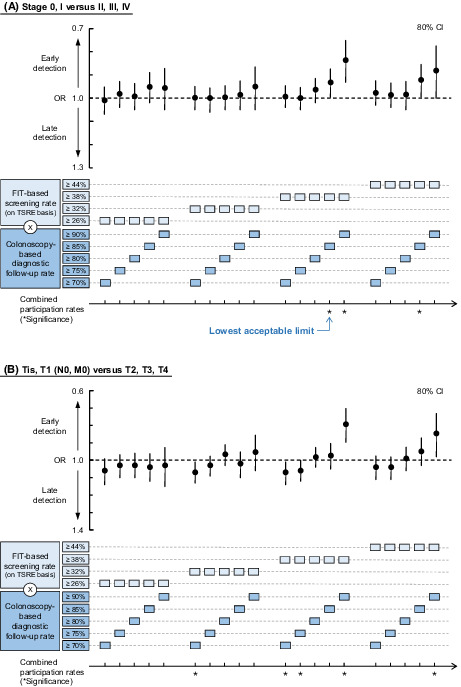

## LAY SUMMARY

- Colorectal cancer screening requires pandemic‐resilience to avoid long‐lasting shutdown.
- This study revealed that, for pandemic‐vulnerable older populations, participation rates ≥38% for the primary screening and ≥ 85% for the follow‐up should be achieved, even during pandemics, to sustain early detection of colorectal cancer.
- This can help pandemic‐resilient target setting by serving as a foundation for balancing the resources between cancer screening and pandemic measures, or a minimum target for shifting to a stool test focused program.

## 1 INTRODUCTION

Colorectal cancer screening (CRCS) was disrupted during the COVID‐19 pandemic in 2020.^1^, ^2^, ^3^ Globally, the decline in screening rates ranged from 28% to 100%.^4^ The impacts appeared greater for regions where screening programs were paused for longer periods of time, and for pandemic‐vulnerable segments of society, such as older populations.^5^ Given that colorectal cancer (CRC) is the second most deadly cancer worldwide, it is vital to build pandemic‐resilient CRCS, especially for vulnerable populations.

Our approach was to clarify the lowest acceptable limit for the screening rate. It was expected that the lowest limit screening rate indicates what percentage of screening rate should be achieved (to what extent screening rate can be compromised) during pandemic. The lowest limit for screening rate by fecal immunochemical test (FIT) can also suggest what level of screening rate is necessary for shifting to FIT‐focused program effectively. In addition, we recognize that the older population is the segment requiring urgent intervention. This is because: (1) healthcare access is disproportionately affected in this population (the COVID‐19 case fatality rate of 9.7% in those aged ≥70 years^6^ could have led to a 5‐year low per capita medical expenditure specifically for those aged ≥75 years in Japan^7^); (2) CRCS backlogs for those aged ≥70 years increased considerably during the 2020 pandemic. In the municipal‐run CRCS program in Japan alone, the screening completion fell by 405,571 from 2019 to 2020^8^; (3) delayed CRC diagnosis is more critical in them^9^; (4) the importance of cancer prevention is still insufficiently focused in this group^10^, ^11^ (those aged >76 years are beyond the CRCS eligibility limits in most countries, despite the recommendation of up to 85 years in the United States^12^ and the full eligibility in Japan); and (5) the impacts on CRC incidence (and, hence, medical expenditure) is substantial in this age group (those aged ≥70 years account for 58.9% of CRC incidence in Japan^13^).

To clarify the lowest limit, a threshold screening rate needs to be identified, above which early detection of CRC is achieved at the area level. However, no studies have specifically examined a threshold screening rate owing to the limited attention of this viewpoint, until the COVID‐19 pandemic. Among a few related studies, Smith et al.^14^ reported that a screening rate of 25.04% did not differ in stage distribution (stage I–II vs. III–IV) between screening and clinically detected patients with CRC aged 50–74 years. Levin et al.^15^ reported that a rise in stool test screening rate of up to 32.0% resulted in a peak of early detection of CRC (earlier than stage III–IV) in patients aged 51–75 years. Although these findings imply the existence of a threshold screening rate for area‐level early detection at stage I–II, the exact value remains unclear and is almost unknown for stage 0–I or for those aged >75 years. Therefore, this study aimed to identify the threshold for CRCS rate that achieves early detection during stage 0–I, focusing on the pandemic‐vulnerable older population as our initial target. To our knowledge, this is the first study to examine pandemic‐resilient target setting for CRCS.

## 2 MATERIALS AND METHODS

### 2.1 Study design

In this nationwide cross‐sectional study, we analyzed the association between area‐level CRCS rates and individual stages at diagnosis. We regarded that the CRCS process has worked as expected when the combined participation in primary screening and diagnostic follow‐up leads to the early detection of CRC during stages 0–I at the area level. Based on this recognition, the threshold participation rate (TPR) was identified as the combination of the lowest screening and follow‐up rates that are significantly associated with early detection.

### 2.2 CRCS program

Japan's population‐based two‐step CRCS program recommends primary screening by a two‐sample annual FIT for healthy, asymptomatic individuals aged ≥40 years (no age limit), and diagnostic follow‐up by colonoscopy for those with a positive FIT result.^16^ CRCS is provided under two types of schemes: mandatory (municipal‐run) and voluntary (employment‐based and private). Municipal‐run CRCS is provided free of charge, employment‐based CRCS is delivered in line with employee welfare benefits, and private CRCS is provided as a for‐profit service. A key challenge in CRCS in Japan is the unachieved target rate, a major cause of the unreduced mortality rate.^8^ As of 2016, among those aged 40–69 years, the primary screening rate was 41.4% compared to the desired level of 50%,^17^ and the follow‐up rate was 69.5% compared to the target of 90%.^18^ Data accessibility is a key issue; complete statistical data are available only for municipality‐managed programs. Therefore, the primary screening rate is not an aggregated value calculated by statistical data but an estimated value based on the national sampling survey.^17^ Data access is even more limited for the follow‐up; there are no accessible national statistical data for voluntarily provided follow‐ups.

### 2.3 Total screening rate equivalent

**FIGURE 1:**
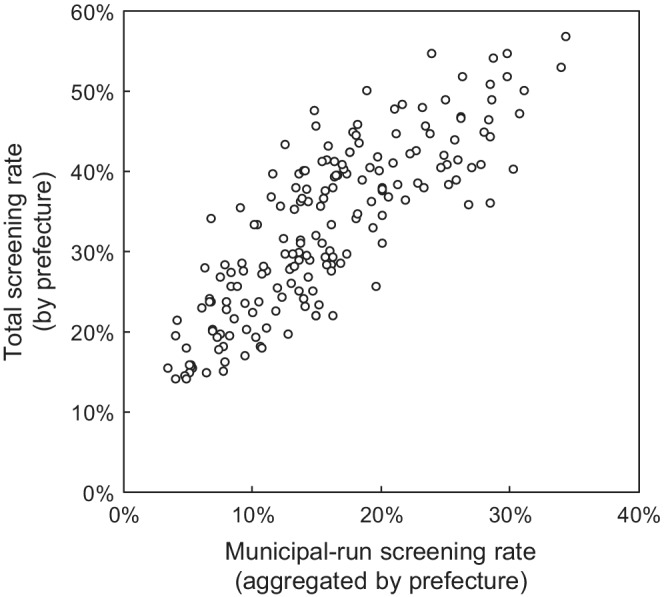
Total screening rate in relation to municipal‐run screening rate. The total screening rates are based on the national sampling survey covering all types of colorectal cancer screening (municipal‐run, employment‐based, and private) for 46 prefectures (excluding Kumamoto, due to an earthquake) by sex and age group (70–79 or ≥ 80 years). Municipal‐run screening rates (by sex and age group) are aggregated by prefecture using the municipality statistical data. A linear relationship is assumed between these two prefectural‐level screening rates (184 observations in total).

A linear regression model was constructed to translate the municipal‐run screening rate to the total screening rate equivalent (TSRE), by comparing the municipal‐run screening rate^18^ and the survey‐based total screening rate^17^ by sex, age group, and prefecture (Figure 1).

### 2.4 Study population

We used an anonymized dataset extracted from the Diagnosis Procedure Combination/Per‐Diem Payment System (DPC/PDPS) database, a medical claim‐based national database.^19^ The DPC/PDPS database includes information on individual patients, such as age, sex, International Statistical Classification of Diseases, Tenth Revision (ICD‐10) code, stage at diagnosis, medical procedures, insurance type, and postal code. As of April 1, 2018, the DPC/PDPS covered 1730 hospitals and 488,563 beds, and Japan's acute inpatients were regarded as almost fully accommodated.^20^ This study included 1165 DPC/PDPS member and 98 nonmember hospitals.

**FIGURE 2:**
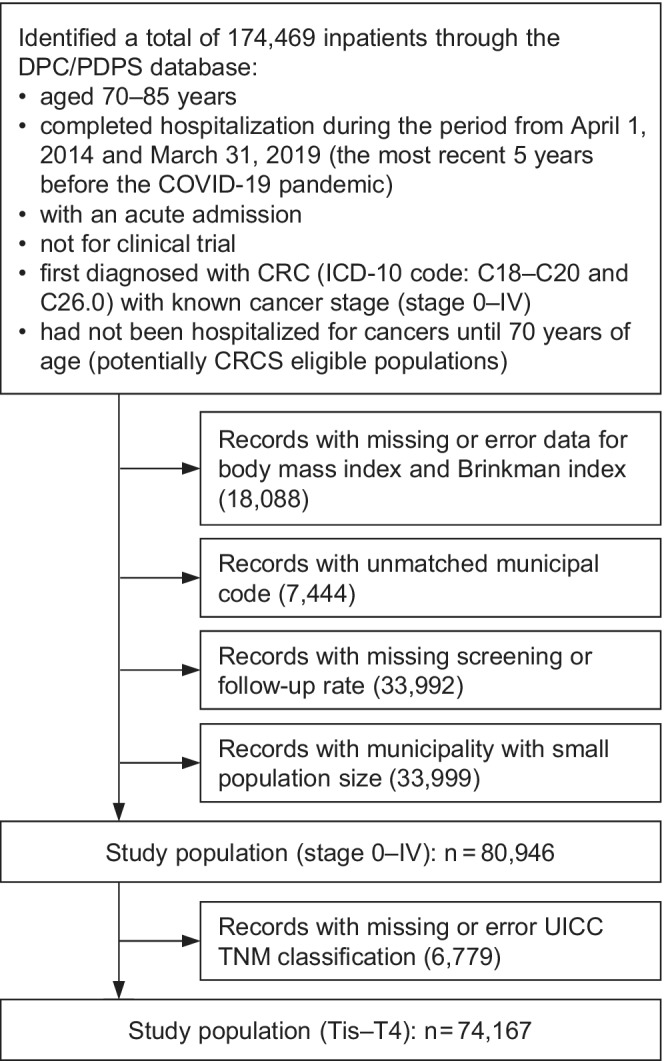
The flowchart of inclusion and exclusion criteria. CRC indicates colorectal cancer; CRCS, colorectal cancer screening; DPC/PDPS, Diagnosis Procedure Combination/Per‐Diem Payment System; ICD‐10, International Statistical Classification of Diseases, Tenth Revision; UICC, Union Internationale Contre le Cancer.

A total of 174,469 inpatients were identified through DPC/PDPS dataset who (1) were aged 70–85 years;^12^ (2) completed hospitalization between April 1, 2014 and March 31, 2019; (3) were first diagnosed with CRC (ICD10 codes: C18.0–C18.9 and C26.0 for colon, and C19.9 and C20.9 for rectal cancer^21^); (4) had not been hospitalized for cancers until 70 years of age as an acute admission; and (5) not partaking in a clinical trial. Excluded patients were those with (1) unavailable data for body mass index (BMI) and Brinkman index (BI); (2) unmatched municipal codes and missing screening or follow‐up rate; and (3) were from municipalities with small population sizes (the number of follow‐up candidates by sex and age group was estimated to be <30). Consequently, 80,946 records (468 municipalities or administrative districts^22^) were extracted for our main analyses (Figure 2).

Selection bias was tested for the stage distribution between the study and excluded populations, because multiple imputation could not be performed, since the proportion of excluded records appeared to exceed the applicability limit.^23^ The sample representativeness of the study population was also evaluated by comparison with the national averages. Upon validation, chi‐square tests were applied to assess the significance of stage distribution.

### 2.5 Primary outcome

The primary outcome of this study was early detection of CRC during stages 0–I. This was mainly because the detection of CRC during stages 0–I, where the survival rate remains over 90% (91.6–94%),^24^ is crucial for mortality reduction. Besides, as the probability of main lymph node metastases remains <1% for these earlier stages,^24^ first‐line therapies (endoscopic or surgical treatment) are less invasive and hence, less costly. Cancer staging was based on the Japanese Classification of Colorectal, Appendiceal, and Anal Carcinoma^25^ developed consistent with the Union Internationale Contre le Cancer staging system.^26^

### 2.6 Variables

**FIGURE 3:**
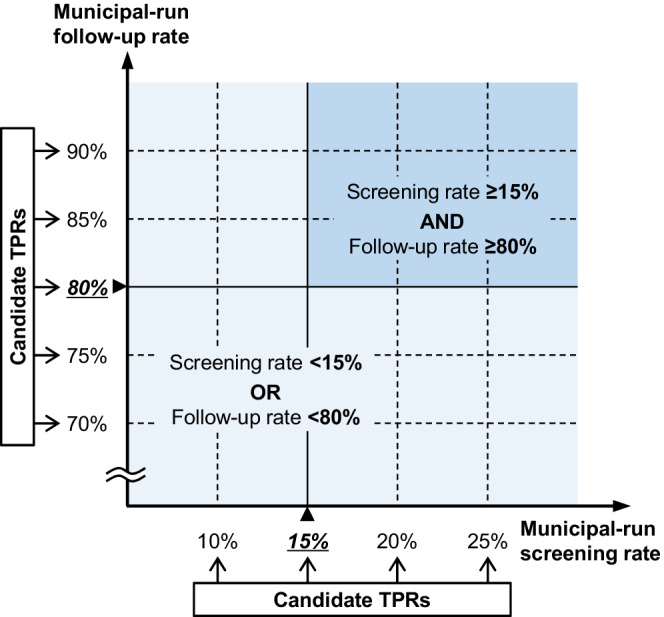
Candidate threshold participation rates (TPRs) and the patterns of combined participation rates in colorectal cancer screening. For identifying the TPR above which early detection is sustained, a spectrum of candidate TPRs are explored, with 10%, 15%, 20%, and 25% for municipal‐run screening rate; and 70%, 75%, 80%, 85%, and 90% for municipal‐run follow‐up rate. Combined participation rate is defined as a binary variable of whether both screening and follow‐up rates are higher than the candidate TPRs, or either of the screening or follow‐up rate is lower than the candidate TPR (illustrated is the case of the candidate TPR for the screening at 15% and for the follow‐up at 80%). A total of 20 patterns of combined participation rates are analyzed. TPR indicates threshold participation rate.

The main explanatory variable was the combined primary screening and follow‐up participation rate. The combined participation rate was dichotomized at the candidate TPRs: 10%, 15%, 20%, and 25% for municipal‐run primary screening and 70%, 75%, 80%, 85%, and 90% for follow‐up (Figure 3). Independent variables included age, sex, cancer site, BMI,^27^, ^28^ BI,^29^ and Charlson comorbidity index (CCI).^30^, ^31^ Other risk factors, such as diet, alcohol consumption, genetic polymorphisms, family history, and past screening behavior and test results were not available in the DPC/PDPS database. As a socioeconomic predictor of health disparity,^32^ individual income level was also used.

### 2.7 Statistical analyses

A multilevel multivariate logistic regression model was used to evaluate the odds ratios (ORs) of the combined participation rate for early detection of CRC at stages 0–I and Tis–T1 (intraclass correlation coefficients were 0.023 and 0.031, respectively). ORs and 95% confidence intervals (CIs) were adjusted for age group, sex, cancer site, BMI, BI, CCI, and income level. We regarded the interaction term (i.e., combined participation rate) to be significant if P for interaction <0.2. The model was tested through sensitivity analyses with assumed area‐level confounders, such as regional healthcare access and employment. Statistical analyses were conducted using R version 4.1.2 and EZR version 1.54.^33^ The dataset was made mainly by Tableau Prep Builder version 2020.3.3.

## 3 RESULTS

### 3.1 TSRE

A linear regression model was constructed to estimate TSRE with an intercept of 0.138 (95% CI [0.117–0.159], *p* < 0.001); coefficient of 1.22 (95% CI [1.10–1.34], *p* < 0.001); and an adjusted *R* ^2^ = 0.68. By adapting this model, the examined TPRs for the primary screening of 10%, 15%, 20%, and 25% were converted to the TSRE‐based values of 26%, 32%, 38%, and 44%, respectively.

### 3.2 Patient demographics

Municipalities with a population ≥ 70,000 (77% of the total population) were included in the study population, which corresponded to an estimated number of follow‐up candidates ≥ 30, using a linear regression model with an intercept: 0, coefficient: 0.435 (95% CI [0.433–0.437], *p* < 0.001), and adjusted *R* ^2^ = 0.59. In testing for selection bias, the differences in the proportion of early‐stage cases were ≤0.6 percentage points between the study and excluded populations (Table S1). Regarding sample representativeness, the average screening rate of the study population was 36.9% (TSRE; 70–85 years) against the national average of 37.0% (70–84 years). Likewise, the obtained average follow‐up rate was 71.1% (70–85 years) compared with the national average of 67.7% (≥70 years). Chi‐square tests applied to the stage distributions (Table 1) showed that higher percentages of stages 0–I cases were constantly observed, when combined participation rates were higher than the TPRs: 38% or 44% for primary screening and 70%, 80%, and 90% for combined follow‐up (32.8–36.0%), compared with the overall percentage of stages 0–I cases (30.9%).

**TABLE 1:** Patient demographics by CRC stage at diagnosis

		Stage 0, I versus II, III, IV						Tis, T1 (N0, M0) versus T2, 3, 4					
		Total	0, I		II, III, IV		*p* ^a^	Total	Tis, T1		T2, 3, 4		*p* ^a^
			*n*	%	*n*	%			*n*	%	*n*	%	
Overall		80,946	25,037	30.9	55,909	69.1		74,167	17,043	23.0	57,124	77.0	
Age^b^	70–79 years	57,723	18,459	32.0	39,264	68.0	<0.001	53,009	12,780	24.1	40,229	75.9	<0.001
	80–85 years	23,223	6578	28.3	16,645	71.7		21,158	4263	20.1	16,895	79.9	
Sex	Male	44,312	14,018	31.6	30,294	68.4	<0.001	40,640	9794	24.1	30,846	75.9	<0.001
	Female	36,634	11,019	30.1	25,615	69.9		33,527	7249	21.6	26,278	78.4	
Cancer site	Colon	55,574	17,450	31.4	38,124	68.6	<0.001	50,735	12,156	24.0	38,579	76.0	<0.001
	Rectum	25,372	7587	29.9	17,785	70.1		23,432	4887	20.9	18,545	79.1	
BMI, kg/m^2^	<21.5	33,496	8787	26.2	24,709	73.8	<0.001	30,295	5853	19.3	24,442	80.7	<0.001
	21.5–24.9^c^	29,968	9887	33.0	20,081	67.0		27,701	6785	24.5	20,916	75.5	
	≥24.9	17,482	6363	36.4	11,119	63.6		16,171	4405	27.2	11,766	72.8	
BI, cigarette year	<800	68,242	21,143	31.0	47,099	69.0	0.459	62,482	14,304	22.9	48,178	77.1	0.197
	≥800^d^	12,704	3894	30.7	8810	69.3		11,685	2739	23.4	8946	76.6	
CCI	<3	64,974	23,124	35.6	41,850	64.4	<0.001	60,723	15,854	26.1	44,869	73.9	<0.001
	≥3	15,972	1913	12.0	14,059	88.0		13,444	1189	8.8	12,255	91.2	
Income level	≥Average^e^	6895	2463	35.7	4432	64.3	<0.001	6320	1694	26.8	4626	73.2	<0.001
	<Average	74,051	22,574	30.5	51,477	69.5		67,847	15,349	22.6	52,498	77.4	
Screening rate (TSRE^f^)	≥26%	60,714	19,414	32.0	41,300	68.0	<0.001	55,540	13,168	23.7	42,372	76.3	<0.001
	<26%	20,232	5623	27.8	14,609	72.2		18,627	3875	20.8	14,752	79.2	
	≥38%	32,835	11,002	33.5	21,833	66.5	<0.001	30,012	7520	25.1	22,492	74.9	<0.001
	<38%	48,111	14,035	29.2	34,076	70.8		44,155	9523	21.6	34,632	78.4	
	≥44%	21,387	7325	34.2	14,062	65.8	<0.001	19,539	4978	25.5	14,561	74.5	<0.001
	<44%	59,559	17,712	29.7	41,847	70.3		54,628	12,065	22.1	42,563	77.9	
Follow‐up rate	≥70%	50,079	15,341	30.6	34,738	69.4	0.020	46,141	10,621	23.0	35,520	77.0	0.744
	<70%	30,867	9696	31.4	21,171	68.6		28,026	6422	22.9	21,604	77.1	
	≥80%	26,719	8081	30.2	18,638	69.8	0.003	24,655	5592	22.7	19,063	77.3	0.173
	<80%	54,227	16,956	31.3	37,271	68.7		49,512	11,451	23.1	38,061	76.9	
	≥90%	5542	1582	28.5	3960	71.5	<0.001	5113	1085	21.2	4028	78.8	0.002
	<90%	75,404	23,455	31.1	51,949	68.9		69,054	15,958	23.1	53,096	76.9	
Combined rate^g^ (TSRE)	≥26% and ≥70%	36,407	11,504	31.6	24,903	68.4	<0.001	33,440	7884	23.6	25,556	76.4	<0.001
	<26% or <70%	44,539	13,533	30.4	31,006	69.6		40,727	9159	22.5	31,568	77.5	
	≥26% and ≥80%	18,291	5752	31.4	12,539	68.6	0.086	16,819	3930	23.4	12,889	76.6	0.175
	<26% or <80%	62,655	19,285	30.8	43,370	69.2		57,348	13,113	22.9	44,235	77.1	
	≥26% and ≥90%	2715	828	30.5	1887	69.5	0.619	2462	542	22.0	1920	78.0	0.247
	<26% or <90%	78,231	24,209	30.9	54,022	69.1		71,705	16,501	23.0	55,204	77.0	
	≥38% and ≥70%	18,003	5913	32.8	12,090	67.2	<0.001	16,585	4058	24.5	12,527	75.5	<0.001
	<38% or <70%	62,943	19,124	30.4	43,819	69.6		57,582	12,985	22.6	44,597	77.4	
	≥38% and ≥80%	8643	2866	33.2	5777	66.8	<0.001	8001	1970	24.6	6031	75.4	<0.001
	<38% or <80%	72,303	22,171	30.7	50,132	69.3		66,166	15,073	22.8	51,093	77.2	
	≥38% and ≥90%	1079	383	35.5	696	64.5	0.001	1003	272	27.1	731	72.9	0.002
	<38% or <90%	79,867	24,654	30.9	55,213	69.1		73,164	16,771	22.9	56,393	77.1	
	≥44% and ≥70%	10,844	3628	33.5	7216	66.5	<0.001	10,018	2479	24.7	7539	75.3	<0.001
	<44% or <70%	70,102	21,409	30.5	48,693	69.5		64,149	14,564	22.7	49,585	77.3	
	≥44% and ≥80%	5324	1802	33.8	3522	66.2	<0.001	4945	1231	24.9	3714	75.1	<0.001
	<44% or <80%	75,622	23,235	30.7	52,387	69.3		69,222	15,812	22.8	53,410	77.2	
	≥44% and ≥90%	688	248	36.0	440	64.0	0.004	635	171	26.9	464	73.1	0.018
	<44% or <90%	80,258	24,789	30.9	55,469	69.1		73,532	16,872	22.9	56,660	77.1	

Abbreviations: BI, Brinkman index; BMI, body mass index; CCI, Charlson comorbidity index; CRC, colorectal cancer; TPR, threshold participation rate; TSRE, total screening rate equivalent.

^a^ Chi‐square tests.

^b^ Dichotomized according to cancer screening behavior (screening rate decreases for aged ≥80 years).

^c^ Defined as the desirable range for aged ≥65 years by the Japanese government.

^d^ Dichotomized according to the risk of CRC incidence.

^e^ The average income level for couple household of active generation estimated by the Japanese government.

^f^ Municipal‐run primary screening rates of 10%, 20%, and 25% are converted to TSRE‐based values of 26%, 38%, and 44%, respectively. Municipal‐run screening rate of ≥30% (50% on TSRE basis) was not analyzed due to insufficient sample size.

^g^ Two‐way interaction between primary screening and follow‐up rate, both dichotomized at the candidate TPRs: 26%, 38%, and 44% for primary screening and 70%, 80%, and 90% for follow‐up.

### 3.3 TPR

**FIGURE 4:**
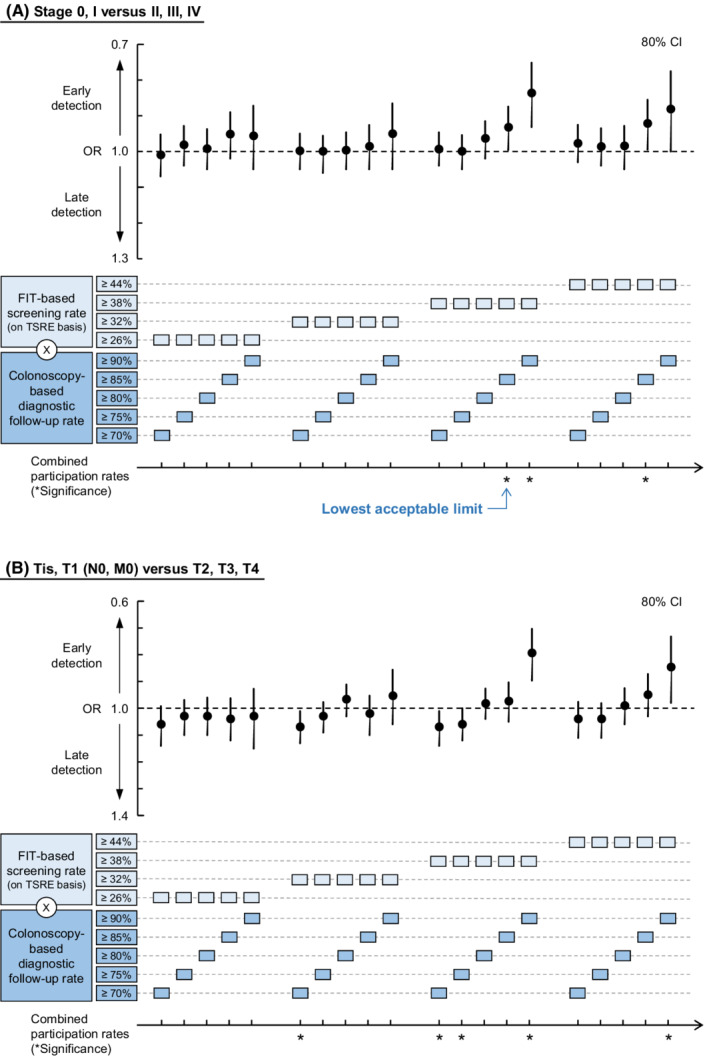
The lowest acceptable limit of the combined participation rates. The association between the combined higher participation rates and the early detection of colorectal cancer (CRC) was evaluated using multilevel logistic regression analysis. An odds ratio (OR) < 1 indicates the early detection of CRC at (A) stage 0–I over II–IV or (B) Tis–T1 over T2–4. The lowest acceptable limit is identified as the combination of minimum primary screening and follow‐up rates that are significantly associated with early detection of CRC. OR indicates odds ratio; FIT, fecal immunochemical test; TSRE, total screening rate equivalent.

With the candidate TPRs of screening rate ≥ 38% and follow‐up rate ≥ 90% (the case with the lowest P for interaction), multilevel logistic regression models (Table 2) revealed that the combined higher participation rates were significantly associated with earlier detection at stages 0–I (OR: 0.84, 80% CI [0.71–0.99], *p* = 0.003) and Tis–T1 (OR: 0.79, 80% CI [0.66–0.96], *p* = 0.002). The analyses for seeking the TPR revealed that the combination of the lowest screening and follow‐up rates that were significantly associated with early detection at stages 0–I was 38% (TSRE) for primary screening and 85% for follow‐up (Figure 4; the data are provided in Table S2). In the sensitivity analyses (Table S3), we obtained the same results with a nonhierarchical model with a 95% confidence level (Figure S1). Further, a similar tendency was observed in the analyses adjusted for the potential confounders, except for some cases, such as those adjusted for municipal population size and income per capita. In the analyses of Tis–T1/T2–4, the significance of early detection at Tis–T1 persisted only when the primary screening rate was ≥38% (TSRE) and follow‐up rate was ≥90%. Moreover, increased ORs (later detection at T2–4) were observed for some cases with the candidate TPR for follow‐up rate at 70% or 75%.

**TABLE 2:** Multilevel logistic regression model for the risk of advanced CRC

	Stage 0, I vs. II, III, IV (*n* = 80,946)			Tis, T1 (N0, M0) vs. T2, 3, 4 (*n* = 74,167)		
	OR	CI^a^	p	OR	CI^a^	p
Age^b^						
70–79 years [ref.]						
80–85 years	1.15	1.10–1.19	<0.001	1.22	1.17–1.27	<0.001
Sex						
Male	0.92	0.89–0.95	<0.001	0.86	0.82–0.89	<0.001
Female [ref.]						
Cancer site						
Colon	0.93	0.90–0.96	<0.001	0.82	0.79–0.86	<0.001
Rectum [ref.]						
BMI, kg/m^2^						
<21.5	1.33	1.29–1.38	<0.001	1.30	1.25–1.35	<0.001
21.5–24.9^c^ [ref.]						
≥24.9	0.89	0.84–0.91	<0.001	0.88	0.84–0.92	<0.001
BI, cigarette years						
<800 [ref.]						
≥800^d^	1.03	0.99–1.08	0.159	1.01	0.96–1.07	0.642
CCI						
<3 [ref.]						
≥3	4.10	3.90–4.32	<0.001	3.72	3.50–3.96	<0.001
Income level						
≥Average^e^	0.86	0.82–0.91	<0.001	0.87	0.82–0.93	<0.001
<Average [ref.]						
Screening rate (TSRE)^f^						
≥38%	0.90	0.86–0.94	<0.001	0.91	0.86–0.96	<0.001
<38% [ref.]						
Follow‐up rate						
≥90%	1.04	0.96–1.13	0.306	1.04	0.95–1.15	0.371
<90% [ref.]						
Combined rate^g^ (TSRE)						
≥38% and ≥90%	0.84	0.71–0.99	0.003	0.79	0.66–0.96	0.002
<38% or <90% [ref.]						

Abbreviations: BI, Brinkman index; BMI, body mass index; CCI, Charlson comorbidity index; CRC, colorectal cancer; TPR, threshold participation rate; TSRE, total screening rate equivalent.

^a^ 80% CI was used for the interaction term (i.e., combined rate) and 95% CI for the others.

^b^ Dichotomized according to cancer screening behavior (screening rate decreased for aged ≥80 years).

^c^ Defined as the desirable range for aged ≥65 years by the Japanese government.

^d^ Dichotomized according to the risk of CRC incidence.

^e^ The average income level for couple household of active generation estimated by the Japanese government.

^f^ Municipal‐run primary screening rates of 20% is converted to TSRE‐based values of 38%.

^g^ Two‐way interaction between primary screening and follow‐up rate, both Dichotomized at the candidate TPRs: 44% for primary screening and 90% for follow‐up.

## 4 DISCUSSION

By defining the older population as a segment requiring urgent intervention in the ongoing COVID‐19 pandemic, we reported here that the screening rate ≥ 38% and combined follow‐up rate ≥ 85% are necessary to sustain the area‐level early detection of CRC (stage 0–I). Below these threshold values, the two‐step CRCS may not perform as expected. Therefore, importantly, it is suggested that the screening rate of 38% and combined follow‐up rate of 85% be regarded as the lowest acceptable limits to be achieved, even during pandemics.

The lowest acceptable limits present a reasonable fit with the currently set target rates. The primary screening rate of 38% was found to be moderately lower than the desired rate in Japan (50%), and considerably lower than the EU and Canadian desirable levels (65% and 60%, respectively).^34^, ^35^ An 85% follow‐up rate lies below the common desirable level in Japan and the EU (90%). These relationships imply that the lowest acceptable limits can help pandemic‐resilient target setting because the participation targets can be made more flexible with a clear, maximum compromise of 12–27% for the primary screening rate and 5% for the combined follow‐up rate to optimize balanced pandemic measures.

Unexpected issues were observed in comparisons with the current screening rates. The threshold screening rate of 38% is comparable with Japan's current screening rate for those aged 70–84 years (37.0%). Also, the 38% rate has not been achieved in some regions, such as France (34.3%, 2008–2009), Czech Republic (22.7%, 2000–2011), Croatia (19.9%, 2007–2011) in the EU,^36^ and Prince Edward Island (33%), New Brunswick (30%), and Newfoundland and Labrador (20.4%) in Canada (2017).^35^ These facts primarily indicate that the lowest acceptable limits might serve as immediate targets for regions where current screening rates are not sufficiently high. Regarding the follow‐up rate, the results require more careful interpretation. Despite the decreased colonoscopy capacity during the pandemic for safety reasons,^4^ the threshold follow‐up rate (85%) is, for some regions, considerably higher than the currently used acceptable level (e.g., 70% in Japan). Although it may be unlikely for follow‐up rates to be higher during a pandemic than in normal times, it is implied that follow‐up rate should not be easily compromised even in such an extraordinary situation. Therefore, feasible approaches should be considered, such as focusing on people with higher risk of advanced CRC (e.g., higher FIT level) or with lower risk of COVID‐19 fatality (e.g., no or few basic diseases). Alternatively, a more rational interpretation might be that follow‐up compliance should be sufficiently enhanced during normal times. Some of the currently used acceptable levels, which might serve not as literal values but as viable milestones toward the desirable rates, might have to be replaced by the lowest acceptable limits.

Interestingly, if our methods are verified and scaled to wider age groups, evidence regarding where the missing true acceptable level should lie may be achieved. EU guidelines^34^ recommend CRCS to achieve a favorable stage distribution in screening‐detected cancers compared with clinically diagnosed cancers. However, the threshold screening rates are not identified. Also, Japan's acceptable screening rates are based more on macro benchmarks, such as actual screening rates in the reference countries, than their influence on stage distribution.

In the discussion of a pandemic‐resilient CRCS, our results may suggest new fundamental elements. Currently examined solutions are mainly regarding: (1) enhancing the participation rate (mailed FIT program^37^ and shift toward a more FIT‐focused CRCS^38^); and (2) improving efficiency for an increased yield (readjustment of the cutoff value of FIT and risk‐stratified colonoscopy based on FIT result^39^). However, solutions for avoiding a shutdown of cancer screening are not explicitly discussed. The lowest acceptable limits might address this missing point. Moreover, for regions with screening colonoscopy‐centered CRCS, such as the United States, the lowest acceptable FIT screening rate might serve as a minimum target in shifting to a FIT‐focused program.

Our findings appeared consistent with, or did not contradict, previous findings. First, regarding stage distribution, the proportion of patients at stage 0–II in this study was 55.6% (70–85 years), whereas in situ and local cancers accounted for 61.6% (≥65 years; 2012–2014) in a cancer registry‐based study by Toyoda et al. (Osaka, Japan).^40^ These values appear roughly consistent, considering the differences in datasets and target regions. Second, given that diagnosis at stages 0–I or Tis–T1 was significantly associated with combined colonoscopy (Table 2), FIT appeared less sensitive for those earlier stages. This observation is supported by previous works.^41^, ^42^ Third, the obtained TPR of 38% (TSRE) for screening and 85% for follow‐up (Figure 4) appeared within the limits of related studies. Evidently, the screening rate of 25.04%, which did not affect the stage distribution in the work by Smith et al.,^14^ is expectedly lower than the TPR of 38%. It is seemingly discrepant that Levin et al. found a peak of early detection when the FIT‐centered screening rate increased to 32.0% (still < 38%) with a follow‐up colonoscopy rate of 72.2% (also lower than the 85%).^15^ Nevertheless, this can be explained by the difference in the definition of early stage. The definition by Levin et al. included stage II, for which FIT has a higher sensitivity. Finally, male sex and higher income level were associated with early detection, consistent with previously reported findings.^40^, ^43^ Because municipal‐run CRCS is free of charge, the association between CRC stage at diagnosis and individual income level is thought to be due to differences in patients' health behaviors rather than a disparity in CRCS access. For further verification, it is expected that our methods are applied to the CRCS eligible population aged <70 years or other cancers using an integrated dataset of area‐level screening rates and individual stage at diagnosis data and that comparisons are made between obtained TPRs and current metrics.

### 4.1 Limitations

This study had some limitations. First, it was a cross‐sectional study; thus, we could not evaluate timeline factors, such as the effect of delayed follow‐up after a positive FIT result.^44^, ^45^ Second, some predictors for CRC incidence were not included, such as diet, alcohol consumption, genetic polymorphisms, family history, and past screening behavior and test results. Third, outpatients were not included, hence a considerable number of potential early‐stage cases could have been omitted in our study, given that endoscopic mucosal resections in outpatient settings accounted for 64.4% and 25.7% for adenomatous polyps <2 and ≥2 cm, respectively (2018).^46^ Underestimated ORs for early detection might have raised the bar for the lowest acceptable screening rates. Fourth, CRCS false positive cases could act as a confounder for the analysis of stages 0–I, because it was not possible to fully exclude T0 cancer, which is regarded as non‐invasive, due to missing TNM classification data. However, since the analyses were focused on inpatients for whom CRC was a main disease in terms of resources utilization, the influence of potential T0 case is thought to be limited. Fifth, our model used area‐level variables along with latent confounders. Based on the findings when adjusted for the assumed area‐level confounders in the sensitivity analyses (Table S3), the model appeared robust for municipal‐level healthcare access indicators (density of hospital, clinic, and gastroenterologist). The model appeared more affected by the variation of municipal population size (≥120,000) and income per capita, indicating that these factors are associated with a higher share of employment‐based or private CRCS, which could undermine the applicability of TSRE. For practical use by public health administrators, further verification is needed. Finally, access to the statistical data of voluntarily provided CRCS was limited. TSRE did not reflect municipal‐level variations of voluntarily provided CRCS. Furthermore, the follow‐up rate remained untranslated because no statistical data were available for estimating TSRE.

## 5 CONCLUSION

CRCS needs to have a pandemic‐resilient target setting option to avoid the long‐lasting shutdown. Our results suggest that, even during a pandemic, CRCS should achieve a primary screening rate ≥ 38% and follow‐up rate ≥ 85% to sustain the early detection of CRC (stages 0–I) in the pandemic‐vulnerable older population. For policymakers, these values suggest the extent to which the CRCS rate can be compromised to balance cancer screening and implementation of pandemic measures and serve as evidence for securing continuity of CRCS during pandemics. The obtained screening rates can also be used as an immediate target or an acceptable level for regions with insufficient screening rates or as the minimum target for shifting to FIT‐focused CRCS in regions with screening colonoscopy‐centered programs. To verify our findings, future studies in varied CRCS settings and eligible populations are warranted.

## AUTHOR CONTRIBUTIONS

Toshiaki Shibata: Conceptualization, data curation, methodology, formal analysis, and writing–original draft. Daisuke Shinjo: Methodology, and writing–review and editing. Junichi Takahashi: Conceptualization and writing–review. Kiyohide Fushimi: Supervision, resources, writing–review, and funding acquisition.

## FUNDING INFORMATION

This study was supported by a Grant‐in‐Aid for Research on Policy Planning and Evaluation from the Ministry of Health, Labour and Welfare (20AA2005).

## CONFLICT OF INTEREST

No relevant financial or nonfinancial interests to disclose.

## ETHICS APPROVAL

This study was approved by the institutional review board at the Tokyo Medical and Dental University.

## PRECIS

Colorectal cancer screening requires pandemic‐resilience to avoid a long‐lasting shutdown. This nationwide study, focusing on pandemic‐vulnerable older population, indicates that colorectal cancer screening should achieve a primary screening rate ≥ 38% and follow‐up rate ≥ 85%, which serves as a foundation for balancing the resources between cancer screening and pandemic measures or as the minimum target for shifting to fecal immunochemical test focused program.

## Supporting information


Supporting Table 1

Supporting Table 2

Supporting Table 3

Supporting Figure 1

## Data Availability

The data availability is not applicable due to an ethical restriction. However, upon reasonable request to the corresponding author, the data can be made available by the DPC research group for researchers who meet the necessary confidential criteria.
