# Supplementary material for: Pandemic‐resilient target setting in colorectal cancer screening for vulnerable older population
Source: Cancer Med. 2022 Jun 5;12(1):619–30. doi: 10.1002/cam4.4907 (PMC9347600; doi:10.1002/cam4.4907)
Supplement: Supplementary file 4 — Supporting Figure 1 [file CAM4-12-619-s003.pptx]

## Slide 1
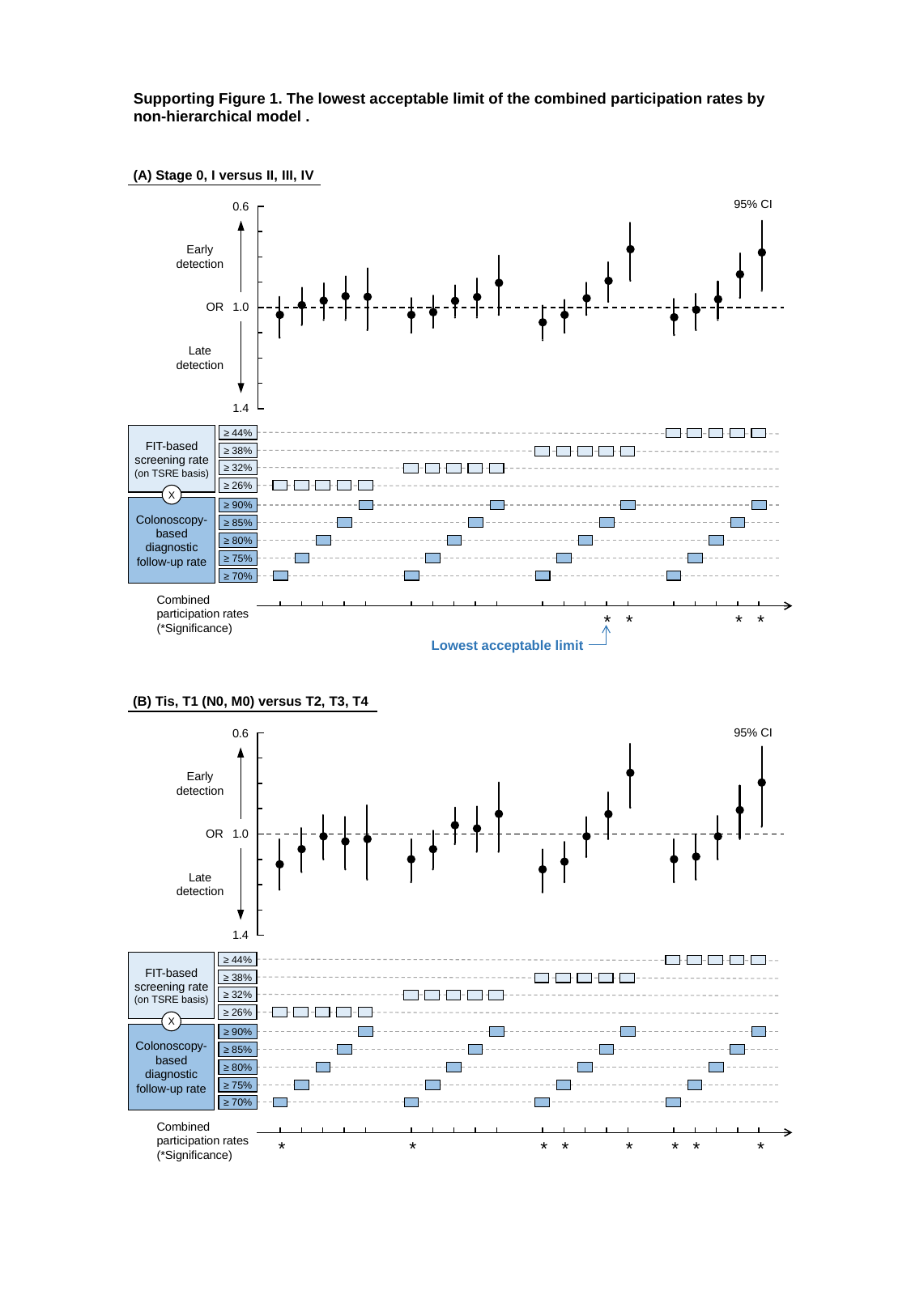

Supporting Figure 1. The lowest acceptable limit of the combined participation rates by non-hierarchical model .
(A) Stage 0, I versus II, III, IV
95% CI
0.6
Early detection
OR
1.0
Late detection
1.4
FIT-based screening rate
(on TSRE basis)
≥ 44%
≥ 38%
≥ 32%
≥ 26%
X
Colonoscopy-based diagnostic follow-up rate
≥ 90%
≥ 85%
≥ 80%
≥ 75%
≥ 70%
Combined participation rates (*Significance)
*
*
*
*
Lowest acceptable limit
(B) Tis, T1 (N0, M0) versus T2, T3, T4
0.6
95% CI
Early detection
OR
1.0
Late detection
1.4
FIT-based screening rate
(on TSRE basis)
≥ 44%
≥ 38%
≥ 32%
≥ 26%
X
Colonoscopy-based diagnostic follow-up rate
≥ 90%
≥ 85%
≥ 80%
≥ 75%
≥ 70%
Combined participation rates (*Significance)
*
*
*
*
*
*
*
*
